# The Power of Gene Technologies: 1001 Ways to Create a Cell Model

**DOI:** 10.3390/cells11203235

**Published:** 2022-10-14

**Authors:** Maxim Karagyaur, Alexandra Primak, Anastasia Efimenko, Mariya Skryabina, Vsevolod Tkachuk

**Affiliations:** 1 Institute for Regenerative Medicine, Medical Research and Education Center, Lomonosov Moscow State University, 27/10, Lomonosovsky Ave., 119192 Moscow, Russia; 2 Faculty of Medicine, Lomonosov Moscow State University, 27/1, Lomonosovsky Ave., 119192 Moscow, Russia

**Keywords:** gene technologies, cell models, cis-/transgene expression, genome editing, CRISPR/Cas9, trans-splicing, primary cell cultures

## Abstract

Modern society faces many biomedical challenges that require urgent solutions. Two of the most important include the elucidation of mechanisms of socially significant diseases and the development of prospective drug treatments for these diseases. Experimental cell models are a convenient tool for addressing many of these problems. The power of cell models is further enhanced when combined with gene technologies, which allows the examination of even more subtle changes within the structure of the genome and permits testing of proteins in a native environment. The list and possibilities of these recently emerging technologies are truly colossal, which requires a rethink of a number of approaches for obtaining experimental cell models. In this review, we analyze the possibilities and limitations of promising gene technologies for obtaining cell models, and also give recommendations on the development and creation of relevant models. In our opinion, this review will be useful for novice cell biologists, as it provides some reference points in the rapidly growing universe of gene and cell technologies.

## 1. Introduction

Modern society faces many biomedical challenges, including epidemics of infectious diseases, age-related diseases, metabolic disorders, and cancers caused by increased lifespan and environmental degradation [1,2,3]. These challenges need to be urgently addressed via the identification of genetic determinants and mechanisms responsible for disease development, the identification of factors correlated with predisposition to different diseases, and the identification of new potential therapeutic targets. Given these challenges, cell models are essential tools for both fundamental and applied biomedical research [4,5].

**Figure 1:**
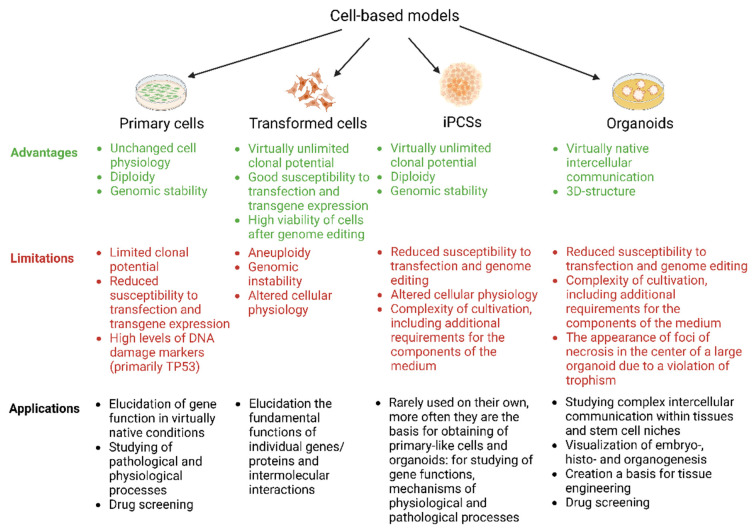
Cell-based models: advantages, limitations, and potential applications. Created with BioRender.com.

Cell-based models represent an in vitro artificial system that allows the simplification and approximation of the physiological and pathological processes taking place in the tissues or organs of living organisms [4,5,6,7]. Cell models have several advantages over animal models, including the simplification of the phenomenon being studied, the fact that experiments can be performed faster and more cheaply, and the possibility of rapid scaling in screening studies with less stringent ethical and legal restrictions (with the exception of human germ cells, zygotes and iPSCs) [8,9]. Cell models are “pioneer” research systems in most fields of biomedicine but are a particularly integral part of the research landscape in the fields of human genetics and regenerative medicine [5,10]. This is the case because cell models permit the study of the functions of individual molecules, of the mechanisms regulating signaling and metabolic cascades, of the pathogenesis of many diseases, and of the cellular machinery responsible for the renewal and regeneration of organs and tissues [11,12,13]. The basis for creating a cell model is founded on transformed cell lines or primary cells isolated from a tissue or organ of a living organism, induced pluripotent stem cells (iPSCs), or multicellular structures (organoids) [14]. Each of these cell culture types has certain advantages, disadvantages, and potential applications, as shown in Figure 1.

The potential of cell models can be drastically expanded via the use of modern genetic engineering approaches that enable researchers to edit specific sections of the genome, including coding DNA, regulatory DNA, and/or RNA, and to vary the expression levels of target proteins. These approaches include cis-/transgene expression in a cell line, genome editing, and trans-splicing. Each of these techniques has both advantages and limitations, which will be discussed and further analyzed below. Such a discussion is valuable because choosing the most appropriate genetic engineering approach at the start of a study allows researchers to obtain quickly the cell model system with the required properties for their research goal.

## 2. Cis-/Transgene Expression in Cell Lines, Genetically Encoded Sensors

**Figure 2:**
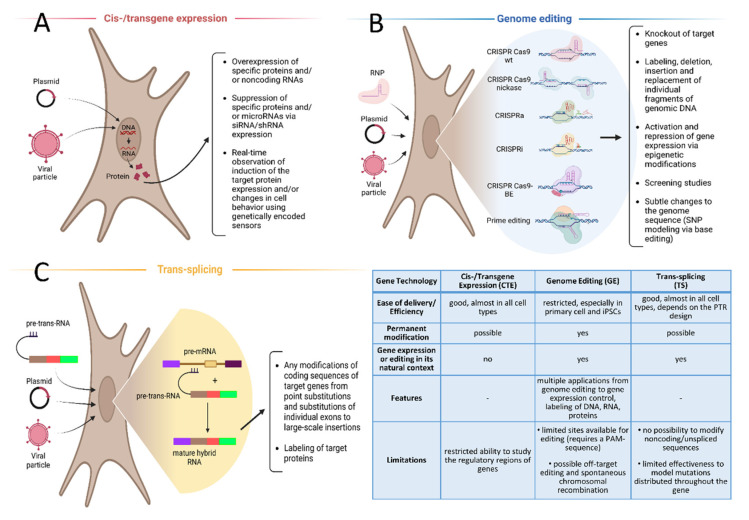
Main genetic engineering technologies for cell-based modelling: principle, advantages, limitations, and potential applications: (**A**)—cis-/transgene expression, (**B**)—genome editing, (**C**)—trans-splicing. Created with BioRender.com.

Cis-/transgene expression is a method of forced ectopic gene expression [15] (Figure 2A) in which the level of transgene expression is greatly determined by the properties of a promoter (versatility, strength, and inducibility) and the type of target cell [16,17]. Cisgene is natural gene from a sexually compatible organism, while the term of transgene comprises all other types of exogenous genes, including artificial and synthetic genes [18].

A promoter is a DNA sequence that controls gene expression; it contains binding sites for transcription factors and RNA polymerases [19]. The number and spectrum of transcription factors involved and the position of their binding sites relative to the transcription start site (TSS) determine the frequency of binding of the transcription complex and, therefore, the efficiency of gene expression that corresponds to such a characteristic of the promoter as “promoter strength” [19,20,21]. The versatility or tissue-specificity of a promoter (the ability to provide gene expression in a wide range of cells or in a certain type of them) is ensured by the presence of binding sites for versatile or tissue-specific transcription factors, respectively [19,20,21]. A constitutively active promoter contains binding sites for transcription factors that are permanently active in a certain cell type, which ensures constant gene expression. An inducible promoter contains binding sites for a transcription factor or transactivator that acquires affinity to the promoter upon the addition of an inducer, that leads to inducible expression of a gene [22].

Cis-/transgene expression can be used not only for therapeutic correction, but also for the creation of cell models. Plasmids—as well as both integrating and non-integrating viral vectors—can be used as transgene delivery vectors, either with or without modification [23,24]. Cis-/transgene expression is widely used both to overexpress specific proteins and/or noncoding RNAs and to suppress them (siRNA/shRNA expression) [25,26]. The main advantage of this approach is the fact that both vector delivery and the expression modification of the target gene (including in primary cells and iPSCs) are highly efficient. The main disadvantage includes the lack of flexibility and the high basal level of gene expression during regular cellular activity. Expression of most genes is tightly regulated depending on the cell type, differentiation status and cell cycle phase [21,27]. All this cannot be reproduced by forced expression of a gene under the control of a strong constitutive promoter. The use of such an approach to study the regulation of gene expression in its natural context is inadequate and is fraught with obtaining unreliable results. For example, it is known that many enzymatic and signaling protein complexes consisting of several components are able to function correctly only at a certain ratio of components, and the imbalance of these components leads to the abnormality of catalysis or signaling [28,29]. Some diseases are caused by misregulated expression of certain genes [28,29].

Therefore, this approach makes it possible to establish the general function of an unknown gene, but it is not suitable for the study of the subtleties of gene regulation, for understanding how pathologies affect gene function, or for the development of promising therapeutic approaches.

To control the expression of a target gene, both constitutive and inducible promoters can be used, and the efficiency of promoters is largely determined by the type of cell line. For instance, many primary cells have the ability to inactivate viral-derived promoters quickly, which can reduce the effectiveness of cis-/transgene expression approaches [30,31].

A newer trend in this technique involves the use of genetically encoded sensors [32,33] that can either be permanently expressed inside target cells or can be induced in response to some signal. Activation of the target signaling cascade or a change in the expression of the target gene (depending on the nature of the sensor) is then manifested by a change in brightness, fluorescence, or in the compartmentalization of a sensor protein. The simplest example of such a sensor is fluorescent protein expression, which is controlled by a tissue- or gene-specific promoter [34,35]. Induction of this promoter, which is usually triggered in parallel with its natural homologue, initiates expression of the fluorescent protein, which permits near real-time observation of induction of the target protein expression and/or changes in cell behavior (e.g., differentiation or transdifferentiation). A smart example of this approach was the MRI-mediated identification of tumor transformation of transplanted stem cells using a ferritin heavy chain (FTH1) reporter under the control of a promoter of the tumor-specific gene progression elevated gene-3 (PEG3) [36].

Significant disadvantages associated with this approach include the delay in the reaction of the sensor in response to promoter induction, differences in degradation dynamics of the fluorescent protein and/or its mRNA relative to the target protein, as well as discrepancies in regulation between the promoter of the sensor and its natural homologue. In the latter case, this can be due to difficulties in modeling the involvement of distant genetic elements (e.g., enhancers, insulators, co-expression with other elements as part of a certain topologically associating domain) during regulation. Significant improvements in the sensitivity and accuracy of sensor proteins can be achieved by introducing the cDNA of a fluorescent protein directly into the structure of the studying gene using genome editing. However, this procedure is difficult, especially when working with primary or hard-to-edit cell cultures (e.g., iPSCs).

Constitutively expressed sensors can be used to detect changes in membrane potential, cell signaling and biochemistry via registration of alterations in certain parameters: sensor brightness, fluorescence specter changes, compartmentalization, that can be quantified [37,38]. These sensors constitutively locate in cytoplasm, cell membrane, or any other cell compartment waiting for the changes that they are encoded to detect. Most of such sensors are based on a fused molecule of a fluorescent protein with a certain functional domain, sensitive for the binding of a target molecule (ion, second messenger, metabolite, enzyme, etc.). Moreover, a prompt way to creation of such sensors was previously described [39]. Sensors for reactive oxygen and chlorine species can be deployed in this way, as can sensors for the activation of PI3K, RAS/ERK signal cascades, and many others [40,41,42]. The main advantages of these sensors are that they are quick to respond to the appearance of target molecules or to activation of the target signaling cascade, and that they have the ability to visualize ongoing changes in different compartments, even within the same cell. 

At the same time, data obtained using such sensors in transformed cells may not correlate with the data obtained in primary cells, a discrepancy that may be due to significant differences in the regulation of pre-mRNA splicing and expression profiles between different cell types [43,44]. For instance, the SPARK (separation of phases-based activity reporter of kinase) reporter, which measures the activity of adenylyl cyclases (PKA-SPARK) and mitogen-activated protein kinase 1 (ERK-SPARK), demonstrated excellent activity in a number of transformed cell lines (HEK, HeLa), according to the literature [42,45] and our own data. More than 50% of these cells responded to the administration of hormones (norepinephrine, PDGF, EGF) by clustering the corresponding sensor (PKA-SPARK or ERK-SPARK). However, an attempt to use SPARK-based sensors in immortalized (ASC52telo, ATCC, #SCRC-4000TM) and primary cultures of human mesenchymal stem cells was unsuccessful. Most cells expressed PKA-/ERK-SPARK sensors at a low level, insufficient for clustering molecules and visualizing signaling, even under the control of strong constitutive eukaryotic EF1a promoter [46]. The reasons for this are unclear, but we assume that this may be due to the significantly larger size of MSCs compared to the size of HEK and HeLa cells (lower concentration of sensor per volume of cell), more strict control over the transcriptional activity of genes (including SPARK sensors) in MSCs, or structural limitations of the SPARK sensor itself. Some MSCs highly expressing PKA-SPARK also did not respond to norepinephrine treatment by sensor clustering, which, as we found, was due to the absence of most isoforms of adenylyl cyclases in these cells [45].

An example of the productive use of constitutively expressed sensor proteins is the family of luciferase-based assays that are used to identify and study genome and transcriptome regulatory sequences (including promoters, enhancers, polyadenylation signals, and microRNA binding sites, among others) [46,47]. The design of some genetic constructs (e.g., the luciferase-bearing pGL3 family of vectors) permits the introduction of potential microRNA binding sites within the coding region as well as within the 5’- or 3’-noncoding regions of luciferase cDNA. This makes it possible to study the subtle mechanisms by which miRNAs influence the expression of individual genes, including by mRNA stabilization, expression activation, or (negatively) RNA interference [48,49].

In general, cis-/transgene expression of a cell line is a fairly effective but rough tool that permits the overexpression of a target protein or microRNA in model cells. A more subtle approach to creating cell models involves genome editing, since this technique changes the nucleotide sequence itself within its natural context.

## 3. Genome Editing

Genome editing (GE) technologies such as the zinc finger nuclease, transcription activator-like effector nuclease, and CRISPR/Cas9 systems, as well as their modifications, collectively possess tremendous potential for creating cell models and the study of the function of proteins, regulatory RNA, and their underlying genes [50,51,52]. GE permits the labeling, deletion, and replacement of individual fragments of genomic DNA, and can also introduce point mutations, knockout target genes, and activate or suppress gene expression, both directly or via epigenetic modification [53,54,55]. The most universal and convenient GE technique of today is the CRISPR/Cas9 system [56] (Figure 2B). Generally, it is a ribonucleoprotein (RNP) complex, consisting of a programmable endonuclease (e.g., Cas9) and a short guiding RNA (gRNA). Replacing of the gRNA alters specificity of the RNP. Many scientific groups took part in the adaptation of the bacterial CRISPR/Cas9 system for GE in Eukarya [56,57,58,59]. The potential of the CRISPR/Cas9 system has been appreciated and the scientists initiating the usage of CRISPR/Cas9 system for GE (Emmanuelle Charpentier and Jennifer Doudna) were honored by the 2020 Nobel Prize in chemistry. 

At present, GE is a crucial technology for the elucidation of pathogenic mechanisms and perspective therapeutic approaches. For example, CRISPR/Cas9 made it possible to establish that LRP1 is one of the main proteins responsible for the capture and distribution of the misfolded tau protein within the brain [60], and to consider neural LRP1 as a potential target for the pathogenetic therapies for Alzheimer’s disease.

Due to its modularity, CRISPR/Cas9 and similar systems permit both whole-genome or restricted screenings of genes involved in particular signaling cascades and biological processes [61,62]. Immortalized transformed cell lines expressing a genome editor or its modification are often used as model objects in such screenings, which restricts the use of this approach in the study of tumor cell biology and in the search for new antitumor therapies [63,64]. CRISPR-based screenings have made it possible to identify MHC I-related protein 1 (MR1), a promising universal target for the highly specific CAR T-cell therapy of solid tumors [65]. However, due to the use of transformed immortalized cells, the data generated by CRISPR screening is of limited utility in understanding the physiological functions of a protein and/or gene. The use of primary cell cultures for CRISPR screening is limited due to their low susceptibility to transduction and the need to use viral particles with a high (20 or more) multiplicity of infection (MOI) [66], which can cause multiple genomic edits, thereby making it difficult to identify the target (edited) gene. This limitation can be partly overcome via genetic modification of iPSCs (using electroporation or lentiviral transduction), followed by their differentiation into the desired cellular phenotype. According to the literature MOI for iPSCs is about 15 [67].

An important feature of genome editing induced by double-strand DNA breaks is that it can be difficult to predict the result, since modifications occurring during DNA repair are determined by the performance of DNA repair mechanisms (e.g., non-homologous end joining (NHEJ), microhomology-mediated end joining (MMEJ), homology-directed repair (HDR)) [68,69], which are in turn determined by cell type and the phase of the cell cycle [70,71]. Thus, according to our data, the seamless removal of a large DNA fragment (~700 bp) using a pair of gRNAs in the HEK293T cell line was observed in more than 30% of alleles. This result demonstrated that in HEK293T, DNA was cut at both gRNA binding sites almost simultaneously, and NHEJ/MMEJ systems were not allowed to repair a single DNA break before the entire DNA fragment was excised [72]. The same approach turned out to be ineffective in an ASC52telo mesenchymal stem line: a pair of gRNAs did not mediate excision of a miRNA-encoded DNA fragment, although sequencing results identified point indels within the cut sites of both gRNAs [72]. Presumably, this was due to a less effective expression of the Cas9 editor in ASC52telo cells compared to HEK293T, which may be compensated for via the use of ribonucleoprotein Cas9*gRNA complexes or by using a powerful inducible transgene expression system [73,74,75].

Some authors have proposed the use of CRISPR-mediated DNA double-strand breaks within miRNA and lncRNA genes to destroy them in order to study their function [76,77,78]. This strategy involves the disruption of lncRNA splicing sites or the hairpin-like miRNA structure via the introduction of indels. In the vast majority of studies, the editing of ncRNA genes is carried out in linear cells [79], and thus the applicability of this method in primary cells has not been established. Moreover, its use is complicated by the difficulty in finding the appropriate PAM and unique gRNA sequence and by the fact that introducing random short indels (the most common outcome of genome editing) may not disrupt the target site. Efficient suppression of miRNA expression in primary cells has been reliably demonstrated only with the use of CRISPR interference (CRISPRi), a modification of CRISPR/Cas9 technology that allows the knockdown of gene expression without introducing a double-strand DNA-break [80].

Furthermore, the introduction of random indels within the region of a double-strand DNA break makes it difficult to obtain the required nucleotide sequence during knockout and HDR modifications, however, they are essential for the CARLIN (CRISPR array repair lineage tracing) technology, which allows the fate of cells in healthy or diseased conditions to be tracked [81]. The NHEJ DNA repair mechanism is also an “ally” of HITI (homology-independent targeted insertion), an approach based on the integration of DNA fragments into DNA double-strand break regions without homology [82]. According to its developers, this approach is quite useful as an alternative to HDR, and its effectiveness was demonstrated by in vivo studies in animals [83]. However, the effectiveness of HITI decreases when using long donor DNAs, since service sequences associated with the DNA-donor are also inserted (in some modifications). Moreover, the HITI approach is not guaranteed to have no extra indels, which reduces the possibility of its use for in-frame insertions into coding sequences [84,85].

When considering new approaches that can be used to create cell models, it is also important to mention that there is a tendency to move from a “rough” gene knockout to modeling more subtle changes within the structure of DNA (i.e., coding and regulatory elements), RNA, or proteins. This approach makes it possible to create more “physiological” cell models that take into account the variety of mutations within a particular gene and allow researchers to identify the molecular mechanisms responsible for a disease that represents new targets for precision therapies.

Existing modifications of GE make it possible to introduce many precise changes in the genome. The greatest efficiency can be achieved when modeling point mutations and SNPs [86,87], as well as establishing the function of proteins and their individual domains. For example, the precision design of gRNAs permitted the establishment of the function of the C-terminal domain of human DUOX2 (dual oxidase 2) in its translocation to the cell membrane and subsequent formation of a functional enzyme transmembrane complex [88]. From our point of view, base editors (CRISPR-BE) provide the most suitable tool for modeling single nucleotide mutations and polymorphisms. However, existing deaminases permit only C- > T and A- > G conversions, and even then, in a rather narrow editing range [86,87,89,90]. This approach can be extended by modifying the PAM-recognizing sequences of existing programmable nucleases [91,92], although this often reduces their accuracy and efficiency, which is apparently mediated by a decrease in helicase activity in such mutants.

An ideal precise genome editing technique would feature the ability to introduce a new sequence with high fidelity or to replace an existing sequence of choice with any other sequence of any arbitrary length. A similar approach, prime editing, was proposed in 2019 by Anzalone et al. [93]. The authors attached a reverse transcriptase to the Cas9 molecule and used a part of the gRNA sequence as a template for reverse transcription in order to make changes within the target DNA sequence. The original version of prime editors had rather low activity and was characterized by a high level of indels [93]. However, a number of modifications proposed further make it possible to increase its efficiency many times over. Optimization of nuclear localization sequence of the genome editor (PE2*) [94], additional single-strand DNA break (PE3) [95], modification of gRNA backbone [96] and blocking DNA mismatch repair mechanisms using transient expression of dominant negative MMR protein (MLH1dn)—PE5 [97]—all increased the modification efficiency of prime editing up to 15–99% (depending on the edited gene, length and nature of the modification) and reduced the probability of indels to 1–10% [96,97].

Another significant limitation of prime editing was the maximal length of possible modification: insertions were limited to 40 base pairs and deletions to 80 base pairs [93]. The latest modifications of prime editing (twinPE) make it possible to overcome this limit and integrate up to 40,000 base pairs into the genome or delete protensive DNA fragments (at least, 800 base pairs long) with an efficiency of up to 80% [98]. In most cases, such high efficiency of editing systems (including prime editing) has been demonstrated on transformed cell cultures: HEK293, HeLa, K562, and U2OS [93,94,95,96,97,98], whereas the efficiency of most genome editors in primary cells and iPSCs remains rather modest. The effectiveness of the latest modifications of prime editing in iPSCs and primary cell cultures has yet to be established, but preliminary studies demonstrate the high potential and flexibility of this editing system.

Genome editing techniques can be harnessed not only to modify the DNA/RNA nucleotide sequence, but also to study the epigenetic regulation of genome functioning [99,100], label DNA and RNA sequences in order to map the genome [101], and enrich the target in particular nucleic acid sequences [102]. The CRISRP/Cas9-based CARPID system (CRISPR-assisted RNA-protein interaction detection) [103] provides unique opportunities for labeling of RNA-binding proteins for subsequent study. This can enrich our understanding of the mechanisms involved in the post-transcriptional modification of RNA and the non-template role played by RNA in the cell. In general, these approaches are based on the use of a dead Cas9 molecule, which acts as a navigator that is associated with a certain functional (modifying) domain. Together, achieving significant efficiency requires multiple targeting (5–6 gRNA) of the DNA or RNA sequence [99,100,101,102,103].

GE permit the permanent modification of DNA/RNA sequences, including in noncoding/regulatory regions of the genome (which distinguishes GE favorably from siRNA/shRNA approaches). However, GE has several limitations, including moderate efficiency, especially in HDR-mediated modifications in primary cells and a significant probability of non-targeted DNA modification [104]. Some of these shortcomings can be overcome by using trans-splicing approaches.

## 4. Trans-Splicing

Trans-splicing is a natural mechanism for splicing several pre-mRNAs into a single mature mRNA (Figure 2C). This mechanism was first discovered in protozoa of the genus Trypanosoma, which use trans-splicing to regulate expression of their own genes [105,106]. Later, trans-splicing in mammalian cells was also discovered; trans-splicing was found to be involved in the formation of one of the isoforms of ESR1 mRNA and in the maturation of some long noncoding RNAs [107]. Moreover, many attempts were made to develop trans-splicing-based transgene expression approaches to treat hereditary diseases and cancers [108,109]. These approaches involve the use of an externally introduced genetic construct encoding a corrected copy of a gene, in whole or in part (the so-called pre-trans-RNA, PTR) [110]. The advantages of this approach include the small size of the genetic construct encoding the PTR, the high efficiency of its delivery, and the high degree of concordance between the formation of a specific trans-splicing product and the expression of the target gene. This makes it possible to simulate the result of gene editing in its natural context with much greater efficiency [111].

Trans-splicing approaches permit us to simulate any modifications of the coding part of target genes, from point substitutions and substitutions of individual exons to large-scale insertions and labeling of target proteins. At the same time, trans-splicing is less efficient than cis-splicing and often leads to the formation of non-physiological mRNA splice forms and proteins [112,113]. These problems, specific to trans-splicing, limit its potential as a transgene expression approach, but do not limit its use as a convenient approach for the creation of cell models. We suggest that optimization of the PTR design, co-expression of siRNA/shRNA to cis-RNA forms, and/or use of modified snRNAs (i.e., for direct splicing), can significantly increase the efficiency and specificity of trans-splicing [114,115]. A thorough study of trans-splicing mechanisms and their optimization may be key for the further development of methods for the therapeutic correction of hereditary diseases, including its application throughout entire postnatal organisms.

In conclusion, here we have considered three main cutting-edge genetic engineering techniques designed to modify the sequences and/or expression levels of target genes in order to study their function in physiological and pathological conditions. Each is characterized by its own advantages and disadvantages (Figure 2), and the choice of a certain technology for creating a cell line is largely determined by the nature of the cells and the type of desired modification.

## 5. Creating Cell Models Based on Transformed, Primary, or Induced Pluripotent Cells

The creation of cell models based on transformed cell lines is quite simple: they have a high proliferative potential (allowing us to obtain clonal populations), suppressed expression of DNA damage markers (to prevent cell death after massive DNA cleavage), and high susceptibility to transfection and transgene expression [116]. However, significant limitations on the use of these cell lines include aneuploidy and genomic instability, as well as substantial differences in cellular behavior relative to the physiology of untransformed cells [117]. In general, such cell lines can only be used to elucidate the functions of individual genes and intermolecular interactions without linking them to cell physiology or to the search for promising drugs. There are many examples of non-physiological behavior in transformed cells, but similar data have been obtained for conditionally primary cell cultures immortalized by telomerase reverse transcriptase hyperexpression. For example, diploid ASC52telo mesenchymal stem cells isolated from adipose tissue were found to have an aptitude for osteogenic differentiation and a decreased adipogenic potential relative to initially isolated MSCs [118]. However, most cells growing in vitro in 2D cultures for an extended period of time gradually lose or change their properties, which may result in radically different transcriptome and differentiation potential.

Efficient genetic modification of transformed cell cultures can be achieved using any of the approaches mentioned above (i.e., cis-/transgene expression, GE, and trans-splicing), and the efficiency of GE can be further increased by reprocessing, as described previously [119]. For the reasons noted above, cell models based on primary cells are especially valuable, since their genome and physiology differ less from the original primary isolated cell culture. This makes it possible to draw more correct conclusions about the role and/or functional mechanism of a given molecule or signaling cascade with respect to physiology and disease. However, it is difficult to modify primary cells due to their limited clonal potential, reduced susceptibility to transfection and transgene expression, and physiologically high levels of DNA damage markers (primarily TP53) [120,121]. Our laboratory has accumulated significant experience in the genetic modification of primary cells (fibroblasts) and conditionally primary cells (ASC52telo cells) using these methods, and we found that cis-/transgene expression (LVP) and trans-splicing proved to be the most effective methods for creating cell models based on these cells. As for GE in primary cell cultures, CRISPR-BE—a cytosine deaminase for creating knockouts (*ADCY1*, *ADRB2* and *ADRB3* genes)—and CRISPR nickase SpCas9D10A (*VDAC1*, *ADCY1* and *ESR1* genes, microRNA genes) were found be the most effective. Conventional SpCas9 was found to be ineffective for introducing mutations into protein genes or for excising DNA fragments (miRNA genes). Potentially, the efficiency of the GE of primary cells can be improved via the use of ribonucleotide-protein complexes (RNPs) by creating high peak-concentrations of them within the nucleus [122,123], via suppression of *TP53* gene expression [124,125], or via GE of primary cells preliminarily dedifferentiated into iPSCs [126,127]. The need for detecting potential off-target activity of GE systems when creating cell models, as well as the tactical approaches to do so, have also been discussed earlier [128].

iPSCs have a number of advantages over primary cell cultures: they have virtually unlimited clonal potential and stable diploid genome, they can be differentiated into almost any cell type, and they can be used for creation of organoids [129]. For efficient genetic modification of iPSCs, the approaches described above for primary cell cultures can be recommended. High clonal potential of iPSCs makes it possible to identify clones with the sought-for genomic variant or modification and expand them to create the desired models. Due to this property, iPSCs are a convenient alternative for solving a number of problems requiring studies on primary cells. The essential question is whether the transcriptome, proteome, and cell behavior of iPSC-derived cells are physiological/natural. To date, there is no simple answer; data is currently accumulating [130,131].

One of the clearest trends in the use of cell cultures is the transition to the supracellular level, which makes it possible to model and study complex intercellular communication within tissues, to study stem cell niches, to visualize embryo-, histo- and organogenesis in 3D cultures, and to create a basis for tissue engineering [132,133]. Depending on the objectives of the study, one or several types of cells can be used as a source for obtaining 3D cultures, including induced pluripotent and/or genetically modified cells, either with or without the addition of matrix proteins [134,135]. Genetically modified cells, primary or iPSCs, for organoid assembly can be obtained using any of the approaches described above.

A significant limitation of such 3D structures is their relatively small size (thickness) and the restricted number of layers, which is due to the absence of capillaries and adequate trophism in the thickness of such structures. The genetic modification of whole organoids using electroporation and viral particles is also possible, although the multilayer nature of an organoid greatly reduces its effectiveness for studying cells in the inner layers [136,137]. Moreover, genetic modification of the whole organoid may cause genetic and phenotypic heterogeneity between its cells, that decreases the quality and reproducibility of the results obtained. At the same time, organoids can be used as models for the development of new approaches and vectors for gene therapy of hereditary diseases (e.g., for the assessment of delivery efficiency).

Techniques for visualizing the structure of such 3D objects (e.g., optogenetics, confocal microscopy, and CLARITY), which are being developed simultaneously, greatly increase the potential of organoid technologies for the future [138,139].

## 6. Conclusions

Despite the active development of animal models, cell-based models are an essential part and, possibly, a predominant tool in experimental biology and regenerative medicine in the near future, since they make it possible to establish the functions of certain molecules and genomic variants, and to determine the mechanisms of action of prospective drug treatments by simplifying the complex systems involved in whole organisms [11,12,13]. Cell models serve as convenient tools for the initial verification of a scientific hypothesis, and the ability to intervene in the operation of living (!) systems on almost any level (due to the co-development of gene editing and imaging techniques) provides unprecedented new opportunities for biomedicine.

In this review, we described the main cutting-edge approaches used for gene modification of cell cultures, including their advantages and limitations. None of the described approaches is universal and cannot be unconditionally recommended for the creation of model cell lines since the required efficiency and accuracy are largely determined by the nature of the object and the type of modification desired. The most complex objects for genetic modification are primary cells, iPSCs, and assembled 3D structures. In our opinion, the most effective and promising techniques for the development of new cells lines are cis-/transgene expression and trans-splicing, along with GE technologies such as the CRISPR-BE and prime editing system or variants of ribonucleoprotein complexes (SpCas9wt, SpCas9 nickase, LbCpf1) [121,122]. Data obtained on primary cell models are the most relevant to the processes taking place in a living organism under both physiological conditions and disease. These models bring us closer to understanding the mechanisms involved in disease progression, tissue renewal, and tissue regeneration. This understanding creates new prospects for the diagnosis, prevention, and treatment of previously incurable pathological conditions.

## Data Availability

Not applicable.
